# Construction and validation of a prognostic model for children with acute upper gastrointestinal bleeding

**DOI:** 10.3389/fped.2026.1678810

**Published:** 2026-03-02

**Authors:** Ruixue Li, Yanmin Wu, Wenting Zhang, Qing Li, Keying Sheng, Yaping Ma

**Affiliations:** 1 Department of Pediatrics, Affiliated Hospital of Jiangnan University, Wuxi, China; 2 Department of Gastroenterology, Affiliated Hospital of Jiangnan University, Wuxi, China; 3 Department of Cardiothoracic Surgery, Children’s Hospital of Soochow University, Suzhou, China; 4 Department of Neonatology, Wuxi Children’s Hospital, Wuxi, China; 5 Department of Neonatology, Wuxi Maternal and Child Health Hospital, Wuxi, China

**Keywords:** acute gastrointestinal bleeding, children, gastrointestinal bleeding, prediction model, prognosis

## Abstract

**Background:**

Gastrointestinal bleeding (GIB) is a common symptom of the pediatric digestive system, with acute upper gastrointestinal bleeding (AUGIB) being extremely dangerous for children. In the present study, we established a risk prediction model for the prognosis of children with AUGIB and provided a new method for early identification of poor prognosis, thereby reducing the disease burden.

**Methods:**

Binary logistic regression analysis was conducted to identify independent risk factors influencing the outcomes of children with AUGIB. The receiver operating characteristic curve (ROC) was generated to assess the predictive efficacy of these risk factors. A nomogram prediction model was constructed, and its performance was evaluated using the consistency index (C-index) and calibration curve. Decision curve analysis (DCA) was applied to estimate the clinical benefits of the intervention.

**Results:**

A total of 372 children who were diagnosed with AUGIB and met the inclusion criteria were enrolled in the study. Neutrophil to leukocyte ratio (NLR), platelet to lymphocyte ratio (PLR), hemoglobin (Hb), high-sensitivity C-reactive protein (hsCRP), and activated partial thromboplastin time (APTT) are independent influencing factors for the outcomes of AUGIB in children. The nomogram model was constructed by including the above independent influencing factors; the consistency index was 0.945 [95 confidence interval (CI): 0.931–0.959]. The DCA was used to assess the prediction performance of the model to obtain net clinical benefits.

**Conclusion:**

A preoperative serum test was an effective and objective method to predict the prognosis of children with AUGIB. The established prognostic risk prediction model had a good prediction effect; it could provide a reference to clinically assess the risk of poor prognosis in children with AUGIB.

## Introduction

Gastrointestinal bleeding (GIB) is a common and relatively critical disease in children. The common causes include reflux esophagitis, rupture of esophageal varices bleeding, and peptic ulcers. The common signs and symptoms of GIB in children include hematemesis (73%), melena (21%), and coffee-ground vomiting (6%) (1). In addition, several causes of GIB have been reported in children, such as gastrointestinal ulcers, varicose veins, intestinal obstruction, intussusception, hemangioma, and other conditions (2, 3). Although the majority of patients have a good prognosis after treatment, without timely intervention, pediatric GIB may also have serious consequences. A study had reported the incidence of GIB to be approximately 6.4%, with a global mortality rate ranging from 5% to 15% (4).

The simple ratio between neutrophil and lymphocyte counts (NLR) measured in the peripheral blood reflects a novel inflammatory indicator of neutrophil and lymphocyte homeostasis (5). The NLR ratio has been used as a surrogate marker of endothelial dysfunction and inflammation in different populations, with prognostic and predictive values (6, 7). The platelet-to-lymphocyte ratio (PLR) is a commonly used blood index to predict and diagnose different diseases. It has emerged as a potentially sensitive marker of inflammatory response (8–10). Sun (11) and Yang (12) used the above new inflammatory indicators to successfully construct a risk prediction model for GIB in children with abdominal Henoch–Schonlein purpura (HSP), and confirmed NLR and PLR as significant predictors. In addition, the construction of prediction models for acute GIB has been widely discussed in the adult population, and the prediction models for bleeding (13), re-bleeding (14), and mortality risk (15) have been successfully constructed, with good clinical predictive values. At present, the research on risk prediction models for acute upper gastrointestinal bleeding (AUGIB) in children is still in its preliminary stage. For example, most models are based on a single radiological finding and do not integrate serological measures. At the same time, insufficient data integrity and cross-center heterogeneity further affect the reliability and applicability of the model. In terms of performance, the existing models still have room for improvement in discrimination and calibration, and often do not fully consider the integration needs of the clinical actual workflow, which limits their practical application value. Therefore, it is necessary to pay more attention to pediatric GIB, identify high-risk groups in time, and provide corresponding treatment to improve clinical outcomes. In the present study, we provided a basis for the medical staff to judge the outcomes of children with GIB in a timely and effective manner and provided a basis for targeted prevention and intervention.

## Methods

### Research participants

We collected clinicopathological data from all children diagnosed with AUGIB at the Affiliated Hospital of Jiangnan University between October 1, 2022, and October 1, 2024, China, who met the inclusion criteria.

Inclusion criteria were (1) age of 6–13 years old, (2) primary clinical manifestations, such as hematemesis, melena, or abdominal pain, with or without peripheral circulation disorders (dizziness, palpitations, amaurosis, transient syncope, shock, etc.), and the diagnosis of AUGIB was confirmed according to the results of endoscopy and laboratory tests, and (3) complete case data.

Exclusion criteria were (1) bleeding caused by systemic diseases such as disseminated intravascular coagulation (DIC) and hematological diseases, (2) patients with incomplete clinical data and laboratory test results, and patients required to be discharged during the treatment.

### General information questionnaire

The general data and clinicopathological data of all patients were collected. The general information included gender, age, body mass index (BMI), clinical manifestations, etiology, admission heart rate, *Helicobacter pylori* infection, family history of GIB, and history of a bad diet (including spicy, greasy, and hot food). Blood routine, blood biochemistry, and coagulation function were examined after admission. These included blood routine (white blood cells [WBC], neutrophils [NEU], lymphocytes [LYM], monocytes [MON], eosinophils [EO], red blood cells [RBC], hemoglobin [Hb], platelet [PLT]) and blood biochemistry (creatinine, urea, albumin, alanine aminotransferase, aspartate aminotransferase, activated partial thromboplastin time [APTT], prothrombin time [PT], international normalized ratio [INR], and D-dimer [DD]). NLR, PLR, and Lymphocyte-to-Monocyte Ratio (LMR) were calculated as follows:

NLR = NEU/LYM,

PLR = PLT/LYM, and

LMR = LYM/MON

### Determination of outcomes

Poor outcome was defined as death, re-bleeding, and more than 3 days in the intensive care unit. Death was defined as all-cause death in the hospital or within 30 days. Re-bleeding was defined (16) as the occurrence of one or more of the following conditions after 48–72 h of no active bleeding or hemodynamic stability confirmed by endoscopy: ① Changes in the shape or frequency of melena or hematemesis, ② bright red blood was withdrawn from the gastric tube, ③ continuous decrease in Hb levels and RBC count, and ④ re-bleeding confirmed by endoscopy.

### Statistical analysis

SPSS 26 (IBM SPSS, USA) was used for statistical analysis. The categorical data of the baseline part are described as frequency and percentage (%). The data were simultaneously divided into two groups according to the “prognosis” results. The Kolmogorov–Smirnov test was used to assess the normal distribution of the measurement data. Categorical data were compared and described using the *χ*^2^ or Fisher's exact test. The *t*-test or Mann–Whitney *U*-test was used to compare the two groups. The difference factors were included in binary Logistics regression analysis, and multicollinearity diagnosis was used to determine whether there was a high linear correlation between independent variables. The final key factors were used to construct the nomogram. The R 4.1.0 software was used to analyze the data, construct a nomogram, calculate the corrected C-index and draw a calibration chart, evaluate the calibration degree of the model, and conduct the Hosmer–Lemeshow goodness of fit test and external verification to evaluate the overall model.

## Results

### Baseline data

The study included 372 pediatric patients, with demographic and laboratory data collected through questionnaires and systematic review. All patients were allocated to either a training set (*n* = 257) from the Affiliated Hospital of Jiangnan University or a validation set (*n* = 115) from the Children's Hospital of Soochow University.

There were 57 patients with poor prognosis in the training set and 29 patients with poor prognosis in the validation set. In the training set, the baseline characteristics of children were analyzed using the t-test or chi-square test. The study included 134 males and 123 females, and the average age of children in the good prognosis group was 9.08 ± 1.90 years. The mean age of children in the poor prognosis group was 9.18 ± 2.06 years. No significant differences were observed in gender, age, BMI, etiology, clinical manifestations, *H. pylori* infection, and heart rate at admission between the two groups (*p* > 0.05). Eight children (4.0%) in the good prognosis group had a family history of GIB, and 50 children (25.0%) had a history of poor diet. Seven children (12.3%) had a family history of GIB in the poor prognosis group, whereas 22 cases (38.6%) had a history of poor diet, with a significant difference between the two groups (*p* < 0.05), as shown in Table 1.

**Table 1:** Baseline data.

Item *n* (%)	Well prognosis	Poor prognosis	*t/X^2^*	*p*
Sex			0.148	0.700
Male	103 (51.5)	31 (54.4)		
Female	97 (48.5)	26 (45.6)		
Age	9.08 ± 1.90	9.18 ± 2.06	0.328	0.743
BMI (kg/m^2^)	21.14 ± 2.27	21.23 ± 2.64	0.249	0.803
Clinical signs and symptoms			1.289	0.732
Hematemesis or blood in the stool	61 (30.5)	18 (31.6)		
Celialgia	63 (31.5)	14 (24.6)		
Nausea and vomiting	43 (21.5)	13 (22.8)		
Other	33 (16.5)	12 (26.7)		
Etiology			1.113	0.291
Ulceration	114 (57.0)	28 (29.1)		
Non-ulcerative	86 (43.0)	29 (50.9)		
Admission heart rate	97.17 ± 19.71	96.30 ± 20.28	−0.291	0.771
Hp. infection			0.001	0.978
Yes	53 (26.5)	15 (26.3)		
No	147 (73.5)	42 (73.5)		
Family history			5.534	0.019
Yes	8 (4.0)	7 (12.3)		
No	192 (96.0)	50 (87.7)		
Poor diet history			4.066	0.044
Yes	50 (25.0)	22 (38.6)		
No	150(75.0)	35(61.4)		

BMI, body mass index.

### Serological comparisons

The serological indicators of the two groups were compared by independent sample *t*-test, and the results are shown in Table 2. Significant differences were noted in PLR, NLR, Hb, hsCRP, BUN, APTT, and PT between the two groups (*p* < 0.05). No significant difference was noted in the blood routine test in the good prognosis group; however, PLR and NLR were significantly lower than those in the poor prognosis group (126.21 ± 9.53 vs. 145.86 ± 11.67; 1.63 ± 0.46 vs. 2.08 ± 0.54). Blood biochemistry revealed that Hb levels in the good prognosis group were significantly higher than those in the poor prognosis group (104.94 ± 19.01 g/L vs. 94.44 ± 19.38 g/L). The levels of high-sensitivity C-reactive protein (hsCRP) and blood urea nitrogen (BUN) were significantly lower than those in the poor prognosis group (3.67 ± 2.28 mg/L vs. 5.67 ± 3.92 mg/L; 5.40 ± 0.81 μmol/L vs. 5.64 ± 0.75 μmol/L). The study of coagulation function demonstrated that APTT and PT levels in the good prognosis group were significantly lower than those in the poor prognosis group (34.16 ± 4.63 s vs. 37.02 ± 4.64 s; 13.44 ± 0.97 vs. 14.03 ± 1.35 s), as shown in Table 2.

**Table 2:** Comparison of serological indexes between the two groups.

Item	PLR	NLR	LMR	PLT (×10^9^/L)	Hb (g/L)	hsCRP (mg/L)	BUN (μmol/L)	Cr (μmol/L)
Well prognosis	126.21 ± 9.53	1.63 ± 0.46	3.21 ± 0.93	238.37 ± 71.34	104.94 ± 19.01	3.67 ± 2.28	5.40 ± 0.81	41.75 ± 9.86
Poor prognosis	145.86 ± 11.67	2.08 ± 0.54	3.21 ± 0.89	236.12 ± 69.61	94.44 ± 19.38	5.67 ± 3.92	5.64 ± 0.75	43.34 ± 9.39
*t*	13.043	6.222	−0.019	−0.210	−3.661	4.892	2.066	1.086
*p*	0.000	0.000	0.985	0.833	0.000	0.000	0.040	0.279
Item	AST (U/L)	ALT (U/L)	ALB (g/L)	APTT (s)	PT (s)	INR	DD (mg/L)	
Well prognosis	23.20 ± 7.85	14.10 ± 3.38	33.69 ± 4.12	34.16 ± 4.63	13.44 ± 0.97	1.27 ± 0.25	0.99 ± 0.41	
Poor prognosis	23.84 ± 7.23	14.17 ± 3.68	33.86 ± 3.98	37.02 ± 4.64	14.03 ± 1.35	1.34 ± 0.24	0.91 ± 0.40	
*t*	0.575	0.129	0.280	4.115	3.728	1.929	−1.205	
*p*	0.566	0.898	0.780	0.000	0.000	0.055	0.229	

PLR, platelet - lymphocyte ratio; NLR, neutrophil/lymphocyte ratio; LMR, lymphocyte/monocyte ratio; PLT, platelet count; Hb, hemoglobin; hsCRP, hypersensitive C-reactive protein; BUN, blood urea nitrogen; Cr, creatinine; ALT, alanine aminotransferase; AST, aspartate transaminase; ALB, albumin; APTT, activated partial thromboplastin time; PT, prothrombin time; INR, international normalized ratio; DD, D-dimer.

### Binary logistic regression of poor prognosis

The parts with different results from the univariate analysis were included in the regression analysis, and the results were shown in Table 3. The regression analysis demonstrated that NLR [odds ratio [OR] = 2.776, 95% confidence interval [CI]: 1.022–7.541], PLR (OR = 1.184, 95%CI: 1.116–1.256), Hb (OR = 0.971, 95%CI: 0.944–0.999), hsCRP (OR = 1.334, 95%CI: 1.022–7.541), and APTT (OR = 1.175, 95%CI: 1.043–1.323) were independent risk factors for poor prognosis (*p* < 0.05), as shown in Table 3. The regression results were diagnosed with collinearity, and the results showed that the tolerance was > 0.8 and the VIF was < 5, indicating that there was no collinearity problem in the results.

**Table 3:** Binary logistics regression analysis of poor prognosis.

Item	B	SE	Wald	*P*	OR	95% CI	
						Upper	Lower
Family history	1.563	0.976	2.563	0.109	4.771	32.319	0.704
Poor diet history	0.482	0.569	0.717	0.397	1.620	4.945	0.531
NLR	1.021	0.520	4.007	0.045	2.776	7.541	1.022
PLR	0.169	0.030	31.480	0.000	1.184	1.256	1.116
Hb	−0.029	0.014	4.028	0.045	0.971	0.999	0.944
hsCRP	0.288	0.100	8.311	0.004	1.334	1.622	1.097
BUN	0.512	0.361	2.015	0.156	1.668	3.383	0.823
APTT	0.161	0.061	7.061	0.008	1.175	1.323	1.043
PT	0.347	0.236	2.154	0.142	1.415	2.249	0.890

PLR, platelet - lymphocyte ratio; NLR, neutrophil/lymphocyte ratio; Hb, hemoglobin; hsCRP, hypersensitive C-reactive protein; APTT, activated partial thromboplastin time; PT, prothrombin time.

### Construction and validation of the nomogram model

**Figure 1:**
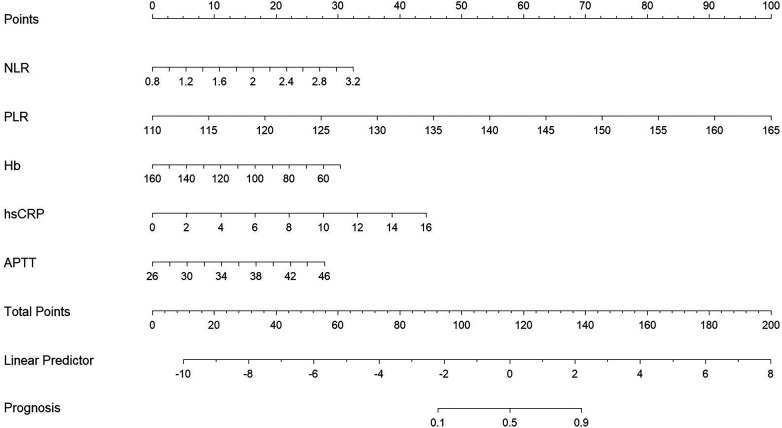
Nomograph.

**Figure 2:**
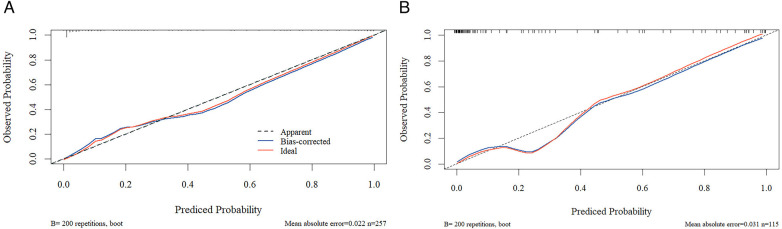
Calibration curve of training set and validation set **(A)** training set; **(B)** validation set.

**Figure 3:**
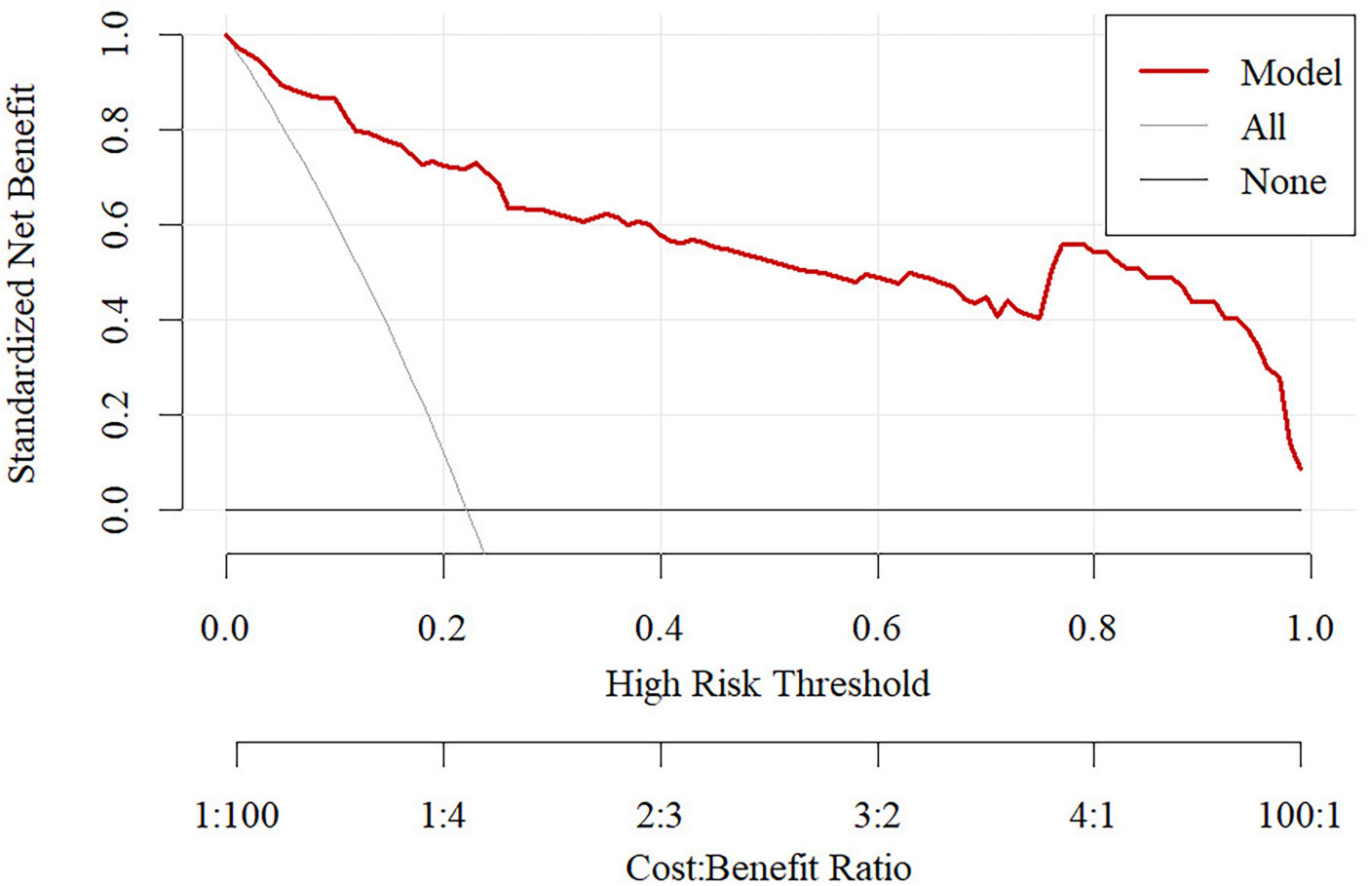
DCA curve.

**Figure 4:**
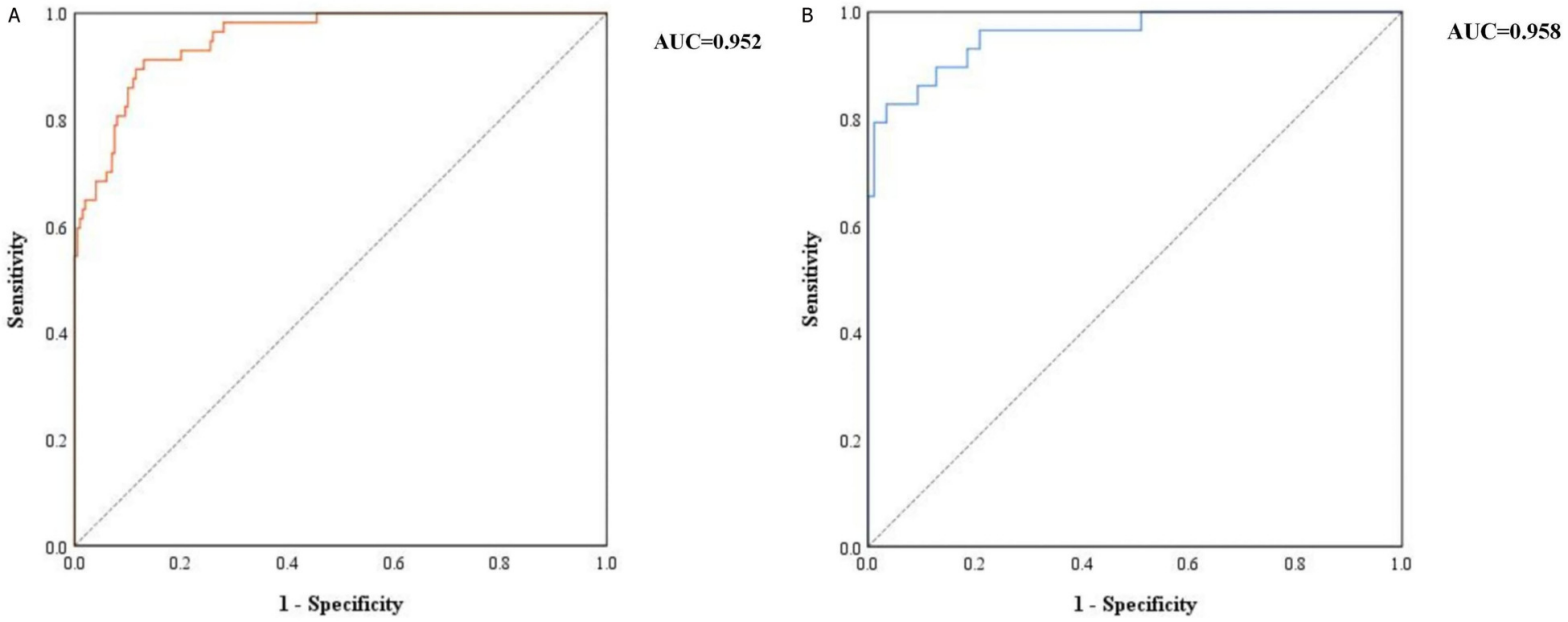
ROC curves of training set and validation set **(A)** training set; **(B)** validation set.

The above independent influencing factors, including NLR, PLR, Hb, hsCRP, and APTT, were included to construct a nomogram model (Figure 1). The consistency index of the training set column line model was 0.945 (95% CI: 0.931–0.959). The prognostic calibration curves of the training and validation sets were close to the reference line, and the column chart model displayed good predictive ability in both the training and validation sets (Figure 2). Decision curve analysis (DCA) was used to assess the predictive performance of the model and obtain net clinical benefits, as shown in Figure 3. DCA curve showed that patients should be closely monitored when the risk value was greater than 20%. The areas under the ROC curves of the training and validation sets were calculated, which were 0.952 (95% CI: 0.925–0.978) and 0.958 (95% CI: 0.917–0.999), as shown in Figure 4.

## Discussion

AUGIB in children is one of the most common clinical emergencies, which can occur at any age. However, only a few studies have provided intensive care statistics related to AUGIB in children (17). For example, the cumulative incidence of AUGIB in the Canadian pediatric intensive care population is 10.2% (18). Furthermore, a prospective cohort study from Thailand that included 110 patients in the pediatric intensive care unit (PICU) requiring mechanical ventilation for over 48 h reported a much higher AUGIB incidence of 51.8%, with 3.6% of cases involving clinically significant bleeding (19). AUGIB in children is characterized by sudden onset and substantial blood loss. The absence of timely treatment could cause shock and even death. A recent study reported the overall mortality rate of UGIB to be 2%, the mortality rate of children newly diagnosed with AUGIB to be 0.37%. However, the mortality rate for severe pediatric cases can be as high as 2.96% (20). Considering these findings and the potential severity of AUGIB in pediatric children, this study aims to identify the risk factors associated with poor prognosis, thereby providing early warning information to guide clinical diagnosis and treatment.

In this study, the demographic characteristics and laboratory data of patients with AUGIB were collected. Univariate and multivariate logistic regression analysis were used to identify risk factors that affect patient outcomes. Higher levels of NLR, PLR, hs-CRP, and APTT and lower levels of Hb were found to be independent risk factors for poor prognosis of AUGIB in children.

Neutrophils play a major role in the inflammatory process and are potential markers for evaluating the diagnosis, severity, and prognosis of different chronic or acute diseases (21, 22). Inflammatory biomarkers such as NLR, SII, and PLR can reflect the systemic inflammatory state and immune response (23). PLR reflects immune balance but specifically emphasizes the involvement of platelets, which are key players in inflammation and endothelial injury. P-selectin is expressed on the surface of activated platelets and binds to P-selectin glycoprotein ligand-1 (PSGL-1) on neutrophils and monocytes. This interaction greatly enhance leukocyte rolling, adhesion, and transendothelial migration, thereby amplifying the inflammatory response. In addition, activated platelets can also release a variety of pro-inflammatory and pro-angiogenic cytokines (such as CD40L, Regulated on Activation, Normal T Cell Expressed and Secreted, Platelet Factor 4, etc.), which directly damage the endothelium or promote the destabilization of atherosclerotic plaques (24). Its pathophysiological mechanism could be related to the increase in the number of neutrophils in the acute phase induced by the gastrointestinal disease itself (25). These increased neutrophils can then recruit various cytokines and chemokines to initiate an inflammatory response, thereby directly damaging the endothelial cells and leading to GIB (26, 27). In addition, activated neutrophils release intracellular substances to the outside of the cells and hydrolyze intercellular junction proteins, thereby destroying the integrity of endothelial cells and causing blood cell leakage (28). Mullady et al. (29) demonstrated that a high number of neutrophils could be associated with increased concentrations of various proinflammatory cytokines, and the severity of bleeding is related to neutrophil-induced immune response and inflammation. In addition, lymphocytes contribute to immune regulation, and abnormalities in their number or function can disrupt this balance, thereby affecting the outcomes. Specifically, a reduction in lymphocyte count impairs the body's capacity to modulate inflammation, which in turn hinders the control of bleeding (30). These points indicate that NLR and PLR could be clinically significant in the treatment and follow-up of patients. hs-CRP participates in the pathophysiological process contributing to AUGIB by activating the complement system, promoting oxidative stress, damaging vascular endothelial cells, and promoting gastrointestinal mucosal injury. High levels of hs-CRP indicate a severe inflammatory response and extensive tissue damage, thus increasing the risk of poor prognosis (31). It is important to note that a key difference between the pediatric and adult immune systems lies in their developmental stages (32). The proportion of naive T cells was high, and the proportion of memory T cells was low in young children (33). Young children have limited neutrophil reserves and have a more intense immune response in the face of inflammatory responses (34). However, there may be little difference between children aged 6–13 years and adults. For hsCRP, levels in healthy children are usually similar to those in adults, generally <3 mg/L, and the median is usually around 0.5–1.0 mg/L. Furthermore, APTT values were significantly prolonged in children compared to adults, especially among neonates and infants aged 1–6 months. These values progressively shortened after 9 years of age, eventually approaching adult levels. However, adult criteria should not be applied directly when assessing inflammation in children. It is necessary to refer to the age-specific reference range provided by the laboratory, combined with clinical manifestations, etiological examination, and other indicators (such as PCT), to make a comprehensive judgment, and more attention should be paid to the serial dynamic monitoring of hsCRP and APTT.

A large meta-analysis (35) identified Hb levels and transfusion requirements as the primary predictors of re-bleeding after AUGIB. Another study demonstrated that early Hb levels may be a predictor of the outcomes of sepsis, and a lower early hemoglobin level could be associated with a worse prognosis for patients with sepsis (36). This suggests the warning effect of Hb on the poor prognosis of patients. The primary function of Hb is to transport oxygen and remove waste. When Hb levels decrease, and cells are starved of oxygen, the organs are severely damaged, the heart is burdened, and immune function is reduced, resulting in an increased risk of infection and poor treatment outcomes.

APTT reflects the sensitivity of the endogenous and exogenous coagulation system and is intricately related to coagulation, anticoagulation, and fibrinolysis. Therefore, we ventured to speculate that the inability to maintain normal coagulation operation when the patient has prolonged APTT could cause re-bleeding after AUGIB, consequently leading to prolonged APTT and generating a vicious circle.

The study by Lan Chen et al. (37) and Yuan L et al. (15) evaluated the outcomes of the risk of death in acute upper GIB, and the main influencing factors included plasma infusion, D-dimer, albumin, potassium, age, red blood cell distribution width, and SpO_2_.This study focused on the risk of bleeding in children. Compared with previous studies, the factors included in this study were not rich enough, and machine learning and other methods were not used for further verification. Additionally, this study had certain limitations. (1) Our definition of poor outcome was primarily based on history, which may have missed certain children. (2) Our study was based only on a retrospective study of hospitalized children. The proportion of AUGIB cases in children in our center relative to the total number of cases in the same period in the local area was not specified, and no attention was paid to screening patients with different visiting times (such as seasons) and different disease severity (such as mild, moderate, or severe bleeding), which may lead to selection bias. Future multicenter or even prospective studies may be needed to help improve the evaluation model. (3) Only logistic regression models were used to screen predictors. In future research, the algorithm should be optimized by first screening variables with Lasso regression and then selecting the optimal model from a range of machine learning methods for verification. Future research can be conducted in a more forward-looking and multi-center manner. It should cover a wider range of geographical areas and population characteristics, reduce selection bias, and enhance its clinical application value. In addition, future studies will include additional variables, including transfusion requirements, more detailed critical scores, treatment, and endoscopic findings in larger multicenter cohorts, with stratification based on disease severity, to further develop and validate a more generalizable advanced predictive model.

## Conclusion

Multiple factors can be used to predict the risk of poor prognosis in children with AUGIB. Moreover, our model can reduce the wrong judgment caused by the subjective assessment and personal experience of doctors, especially for certain primary and community hospitals, and can provide additional options for possible early assessment of poor prognosis.

## Data Availability

The raw data supporting the conclusions of this article will be made available by the authors, without undue reservation.
